# The Late Iron Age in Switzerland: a review of anthropological, funerary, and isotopic studies

**DOI:** 10.1007/s12520-023-01838-w

**Published:** 2023-08-25

**Authors:** Christine Cooper, Marco Milella, Sandra Lösch

**Affiliations:** Department of Physical Anthropology, Institute of Forensic Medicine, University of Bern, Bern, Switzerland

**Keywords:** Burials, Bioarcheology, Funerary rites, Demography, Paleopathology, Stable isotopes

## Abstract

The Iron Age in continental Europe is a period of profound cultural and biological importance with heterogeneous trends through space and time. Regional overviews are therefore useful for better understanding the main cultural and biological patterns characterizing this period across the European regions. For the area of modern Switzerland, a rich archeological and anthropological record represents the Late Iron Age. However, no review of the main anthropological and funerary patterns for this period is available to date. Here we assess the available demographic, paleopathological, funerary, and isotopic data for the Late Iron Age in the Swiss territory, and summarize the cultural and biological patterns emerging from the available literature. Finally, we highlight a series of research avenues for future studies.

## Introduction

### The Late Iron Age in Switzerland: an overview

Normally defined as the time between the eighth century BCE and the territorial spread of the Roman Empire (Table 1), the Iron Age in Continental Europe featured important biocultural changes (Champion et al. 2016; Kruta 2009; Müller et al. 1999; Vitali 2004, 2011; Wells 2011).

**Table 1:** Approximate timeline of archaeological periods in Switzerland, finer subdivisions are only included for the Late Iron Age. The table is adapted after Stöckli et al. (1995); Hochuli et al. (1998); Müller et al. (1999); Flutsch et al. (2002); Windler et al. (2005); Marti et al. (2014); Baeriswyl et al. (2020)

Date	Period	Sub-period	
1500–1900 CE			Post-Medieval/modern period
1050–1500 CE			High/Late Middle Ages
400–1050 CE			Early Middle Ages
50 BCE–400 CE			Antiquity/Roman Empire
80–15 BCE	Late La Tène	LT D2	Late Iron Age
150–80 BCE		LT D1	
200–150 BCE	Middle La Tène	LT C2	
260–200 BCE		LT C1	
320–260 BCE	Early La Tène	LT B2	
400–320 BCE		LT B1	
450–400 BCE		LT A	
800–450 BCE	Hallstatt	Ha C–D	Early Iron Age
2200–800 BCE			Bronze Age
5500–2200 BCE			Neolithic

These included profound social, economic, and political innovations, as well as the development of distinctive forms of artistic expression (Champion et al. 2016; Müller et al. 1999; Müller and Lüscher 2004; Stöckli 2016; Wells 2020). The establishment and development of networks between different regions and cultures led to a substantial flow of ideas and people, a pattern that anticipates the Roman Empire biocultural mosaic. Although featuring important cultural similarities across regions, the Iron Age was also characterized by marked socioeconomic and biocultural heterogeneity with effects on human lifestyle and biology (Kruta 2009; Laffranchi et al. 2019; Laffranchi et al. 2016; Moghaddam et al. 2016; Moghaddam et al. 2018; Scheeres et al. 2014; Wells 2011). We need a contextualized overview of these processes when we try to evaluate the biocultural relevance of the Iron Age and its relationships with preceding and subsequent periods, namely the Bronze Age and Roman Times.

In today’s Switzerland, the first archaeological traces of a settlement attributable to the Early Iron Age date to ca. 800 BCE. This is the case, for example, at Weiler Frasses, near the Lake Neuchâtel (Müller et al. 1999). The Roman occupation of Swiss territories started around 200 BCE in the southern region of Ticino, followed by the southwestern region of Geneva around 55 BCE. Following the Alpine campaign of 15 BCE and up to the fifth century CE, the area of today’s Switzerland was firmly part of the Roman Empire (Müller et al. 1999; Tarpin et al. 2002). The Swiss Iron Age includes an earlier and a later phase (Champion et al. 2016; Kaenel 1999): The Early Iron Age, or Hallstatt period, named after the village Hallstatt in Austria, dates from about 800 to 450 BCE. The Late Iron Age or La Tène period is named after the archeological site at Lake Neuchâtel in Switzerland and dates from about 450 to 15 BCE (Cunliffe 1997).

Archeological sites from the La Tène period, including settlements, *oppida*, cemeteries, and sanctuaries, are numerous in Switzerland (Müller and Lüscher 2004). Given the specific focus of this work, we present in the following overview only funerary contexts for which anthropological results have been published. For more archeologically oriented syntheses, we refer the reader elsewhere (Kaenel 1999; Müller et al. 1999; Müller and Lüscher 2004).

**Fig. 1:**
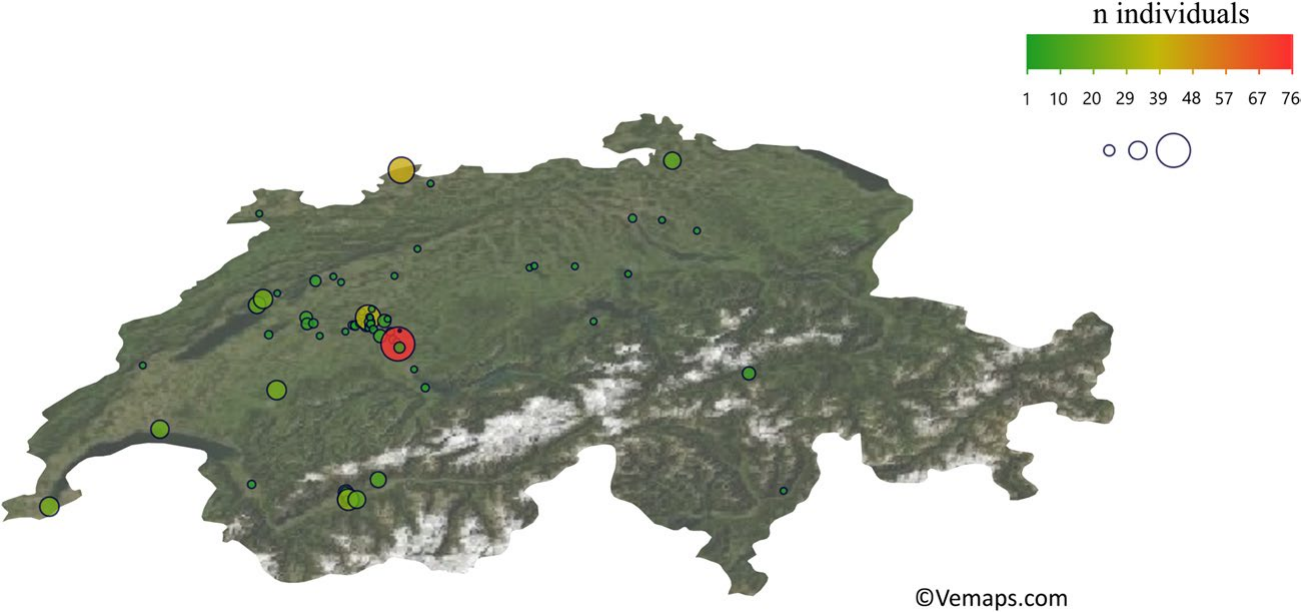
Geographic distribution and numbers of Late Iron Age funerary contexts in the Swiss territory. Outline of modern Switzerland adapted from Vemaps.com

Figure 1 illustrates the geographic distribution of the mentioned contexts.

Only few human remains are preserved from sites in eastern and central Switzerland. In the northeast of the Swiss Plateau in the canton of Zürich, the Andelfingen cemetery probably belonged to a later *oppidum* (Viollier 1912), while isolated graves and small groups were also found (Altorfer and Schmid 1996; Fischer 1994; Horisberger 2019).

The Basel-Gasfabrik site in northern Switzerland, an unfortified proto-urban settlement with two necropolises, was discovered in 1911 and has been under research since (Hüglin and Spichtig 2010; Jud and Spichtig 1998; Knipper et al. 2017, 2018; Pichler et al. 2015; Pichler et al. 2013; Pichler et al. 2014; Rissanen et al. 2013; Schaer and Stopp 2005; Trancik Petitpierre 1996). Both necropolises yielded a total of almost 200 individuals, but it is assumed that many more graves were destroyed during earlier construction without archeological surveillance. Between 2005 and 2007, excavations took place in both necropolises during which 42 skeletons were documented. Human remains were found not only in the two cemeteries, but also within various settlement features (Pichler et al. 2015; Pichler et al. 2013).

In total, eight Late Iron Age sites are known around Luzern in central Switzerland (Nielsen 2014), but human remains are only preserved from few (Lehner 1986; Moghaddam et al. 2013a, 2013b; Nielsen 2014).

Southern Switzerland (cantons of Grisons, Ticino, and Valais) contains many sites (Kaenel 1999); in Grisons, human remains from two sites are preserved. The cemetery in Trun-Darvella at the Rhine headwaters may have belonged to a settlement nearby. Among the grave goods, lances, swords, and costume objects were found (Tanner 1980). The human remains were not preserved and/or collected systematically at the sites in Castaneda (Keller-Tarnuzzer 1933; Nagy 2000; Nagy 2008) and Cama (Reitmaier 2020). In the canton of Ticino, large Celtic cemeteries existed along important trade routes between the southern Alpine valleys and Raetia (e.g., Giubasco or Arbedo), but most were excavated around 1900 and bones were not collected. Due to the characteristics of the soil, only very few human remains from this area are preserved (Costa 2005; Del Fattore 2007). A great number of Late Iron Age burials in the canton of Valais were destroyed by farming activities during the nineteenth century. In the last decades, several sites in and around Sion were excavated professionally and have been subject to comprehensive research. They include assemblages of mostly well-preserved burials in Sion-Sous-le-Scex, some of which were equipped with costume objects and weapons and in Bramois, only 3 km away. It is assumed that a settlement was located nearby or that rituals were performed there (Curdy et al. 2009; Debard 2014). Several more burials were found at various locations in Sion and in Randogne (Curdy et al. 2009; Debard 2014; Hofstetter 2018; Moret et al. 2000).

The highest concentration of graves was discovered in the canton of Bern, in the Swiss midlands. Geographical clusters of findings suggest two centers: one around the Belp Mountain, the other around the Enge Peninsula where the Celtic *oppidum* “Brenodor,” a proto-urban settlement on the territory of today’s federal capital of Bern, was located. This was the focal point of burials nearby (Jud and Ulrich-Bochsler 2014). The largest cemetery is Münsingen-Rain with originally 220 burials (Hodson 1968; Müller 1998; Müller et al. 1999; Wiedmer-Stern 1908). The Aare valley between the city of Bern and Lake Thun seems to have been part of a fairly developed settlement area in the Late Iron Age (Müller 1996).

Important sites like the eponymous La Tène and Cornaux-Les Sauges in the canton of Neuchâtel comprised skeletal findings from the La Thielle River that were associated with objects like vehicle parts, swords, lances, and shields (Kaenel 2007; Müller 2002; Müller et al. 1999; Schwab 1989). Most human remains from the canton of Fribourg are from the cemeteries at Gumefens (Jud 2009), Gempenach (Kaenel and Favre 1983), and Kerzers (Ramseyer 1997). The two cemeteries in Gumefens contained a high proportion of burials with weapons and richly equipped female burials that suggested a high social status of the individuals buried there (Jud 2009).

Noteworthy concentrations of graves in western Switzerland in the cantons of Vaud and Geneva are located along Lake Geneva (Kaenel 1990, 1995; Maroelli and Gallay 2014). Other human remains in this area were found at sites that are interpreted as sanctuaries (Bonnet et al. 1989; Dietrich et al. 2007, 2009a, b; Haldimann and Moinat 1999; Moinat 1993; Simon and Desideri 1999).

As mentioned, detailed anthropological analyses have been performed only for some Iron Age skeletal remains from the Swiss territory. The results of these investigations are often difficult to access due to their publication in local archeological magazines/gazettes in German, French, or Italian language. These works are moreover typically focused on single contexts. To date, a synthesis of the anthropological data for this period and geographic area is still missing, and a comparison with preceding and later chronological phases is absent. This hampers the reconstruction of a larger picture and the exploration for common or diverging patterns (e.g., in demography, life quality, diet, funerary rituals).

Based on these premises, our study aims to review and synthesize the available anthropological data for the Late Iron Age in Switzerland according to a suite of topics. These include (1) the relative representation of skeletal remains; (2) the variability of funerary contexts and funerary rituals; (3) main demographic patterns; (4) paleopathological patterns; (5) paleodietary and paleomobility reconstructions.

## Material and methods

We first screened all available anthropological reports for late Iron Age (ca. 4^th^–1^st^ centuries BCE) contexts from the Swiss territory. We then excluded all contexts without inhumations (e.g., incinerations) and those lacking the minimal anthropological assessment of age-at-death and sex for at least part of the individuals. The final dataset includes 474 individuals from 75 archeological sites covering 12 cantons. The number of individuals from each context ranges from 1 to 76 (Table 2).

**Table 2:** The Late Iron Age sites in Switzerland used in this study. *N* refers to the number of individuals for which anthropological results have been published

Site code	Canton	Site name	Year(s) of excavation	*N*	Dating	Burial type	References
LIA 01	BE	BELP Sonnegg-/Neumattstrasse	1950, 1972, 1977	8	LT C	Regular	(Debard 2014; Hug 1956; Schoch and Ulrich-Bochsler 1987; Suter and Ulrich-Bochsler 1984)
LIA 02	BE	BERN Bümpliz, Turnplatz Statthalter-Schulhaus	1949	1	LT B-C	Regular	(Hug 1956)
LIA 03	BE	BERN Bümpliz, Zypressenstrasse	1954	2	LT B-C	Regular	(Hug 1956; Schoch and Ulrich-Bochsler 1987)
LIA 04	BE	BERN Bernastrasse	1937	1	LT B-C	Regular	(Hug 1956)
LIA 05	BE	BERN Bümpliz, Franken-/Morgenstrasse	1952	3	LT B-C	Regular	(Hug 1956; Schoch and Ulrich-Bochsler 1987)
LIA 06	BE	BERN Bümpliz, Statthalterschulhaus/Morgenstrasse	1952	1	LT B-C	Regular	(Hug 1956)
LIA 07	BE	BERN Bümpliz, Zedernstrasse	1950	4	LT B-C	Regular	(Hug 1956)
LIA 08	BE	BERN Muristalden	1893	2	LT B-C	Regular	(Hug 1956)
LIA 09	BE	BERN Reichenbach-/Rossfeldstrasse (Enge Peninsula)	1950, 1999	39	LT C2/D1	Regular	(Hug 1956; Jud and Ulrich-Bochsler 2014; Ulrich-Bochsler 2010; Ulrich-Bochsler and Rüttimann 2014)
LIA 10	BE	BERN Schärloch (Enge Peninsula)	1932	1	LT	Regular	(Hug 1956)
LIA 11	BE	BERN Spitalacker, Viktoriastrasse	1896	4	LT	Regular	(Hug 1956)
LIA 12	BE	BERN Steigerhubelstrasse 6	1993	1	LT	Regular	(Gutscher and Suter 1994; Ulrich-Bochsler 2010)
LIA 13	BE	BERN Thormannmätteli (Enge Peninsula)	1932	1	LT	Regular	(Hug 1956)
LIA 14	BE	BERN Tiefenauspital (Enge Peninsula)	1925	1	LT	Regular	(Hug 1956)
LIA 15	BE	BERN Weissenbühlweg/Wabernstrasse	1895	4	LT	Regular	(Hug 1956)
LIA 16	BE	BERN Wilerfeld	1857	1	LT	Regular	(Hug 1956)
LIA 17	BE	BOLLIGEN Ferenberg	1884	1	LT	Regular	(Hug 1956)
LIA 18	BE	BÜETIGEN -	1933	1	LT	Regular	(Hug 1956)
LIA 19	BE	BÜETIGEN Griengasse	1970	1	LT B2	Regular	(Schoch and Ulrich-Bochsler 1987; Thommen 1980)
LIA 20	BE	FERENBALM-RIZENBACH Kiesgrube Vogelbuch	1879	3	LT	Regular	(Hug 1956)
LIA 21	BE	IPSACH Räberain	2009	5	LTB2-C1	Regular	(Moghaddam and Lösch 2015; Ramstein 2010)
LIA 22	BE	KEHRSATZ Hungert	1967	2	LT	Regular	(Schoch and Ulrich-Bochsler 1987)
LIA 23	BE	KIRCHLINDACH Niederlindach	1883	1	LT	Regular	(Hug 1956)
LIA 24	BE	KÖNIZ Kiesgrube im Hubackergut	1897	1	LT	Regular	(Hug 1956)
LIA 25	BE	KÖNIZ-WABERN -	1932	1	LT	Regular	(Hug 1956)
LIA 26	BE	MÜHLEBERG-GÜMMENEN Kiesgrube bei Trühlern	1904	1	LT	Regular	(Hug 1956)
LIA 27	BE	MÜNSINGEN Hintergasse	1985	1	LT D1	Regular	(Grütter and Ulrich-Bochsler 1990; Schoch and Ulrich-Bochsler 1987)
LIA 28	BE	MÜNSINGEN Rain	1906	76	LT A-C2	Regular	(Alt et al. 2005; Hodson 1968, 1998; Jud 1998; Moghaddam 2016; Moghaddam et al. 2013a, b; Moghaddam et al. 2015; Moghaddam et al. 2016; Moghaddam et al. 2018; Müller 1996, 1998; Müller et al. 1999; Wiedmer-Stern 1908)
LIA 29	BE	MÜNSINGEN Tägermatten	1930	2	LT	Regular	(Hug 1956)
LIA 30	BE	NIEDERWICHTRACH Seinfeld-Kiesgrube	1904, 1967, 1968	5	LT D	Regular	(Hug 1956; Schoch and Ulrich-Bochsler 1987; Stöckli 1995)
LIA 31	BE	ORPUND Munthel	1913	1	LT	Regular	(Hug 1956)
LIA 32	BE	RUBIGEN Riedacher	1977/1978	2	LT A-B2	Regular	(Schoch and Ulrich-Bochsler 1987; Von Kaenel 1979)
LIA 33	BE	SPIEZ Schönegg	1872	2	LT	Regular	(Hug 1956)
LIA 34	BE	STETTLEN-DEISSWIL Kiesgrube Bühlmann	1936, 1942, 1945	8	LT B1-C2	Regular	(Hug 1956; Rey 1999; Schoch and Ulrich-Bochsler 1987)
LIA 35	BE	THUN-LAUENEN Rosenweg	1972	1	LT C1	Regular	(Schoch and Ulrich-Bochsler 1987)
LIA 36	BE	WIEDLISBACH Mühlackerweg	1977	1	LT C2	Regular	(Schoch and Ulrich-Bochsler 1987; Suter and Ulrich-Bochsler 1984)
LIA 37	BE	WORB-RICHIGEN Stockeren-Kiesgrube	1906–1919		LT	Regular	(Hug 1956)
LIA 38	BE	ZOLLIKOFEN Station	1905	1	LT	Regular	(Hug 1956)
LIA 39	BL	PRATTELN Meierhof	2007	1	LT C1	Regular	(Marti 2008)
LIA 40	BS	BASEL Gasfabrik	1964, 2005–2007	43	LT D1	Regular/irregular	(Pichler et al. 2015; Pichler et al. 2013; Pichler et al. 2014; Rissanen et al. 2013; Schaer and Stopp 2005)
LIA 41	FR	BÖSINGEN Noflen	1974	1	LT C1	Regular	(Gilbert Kaenel 1990)
LIA 42	FR	GEMPENACH Forstmatte	1979	6	LT C1-C2	Regular	(Kaenel and Favre 1983)
LIA 43	FR	GUMEFENS La Perrey/Sus Fey	1978–1980	21	LT B2/C1	Regular	(Jud 2009; Schwab 1989)
LIA 44	FR	KERZERS Vennerstrasse	1995	7	LT B1/B2	Regular	(Menoud and Ramseyer 1995; Ramseyer 1997)
LIA 45	GE	GENÈVE Port, Rue de La Fontaine	1920s	21	LT C2-D2	Irregular	(Bonnet et al. 1989)
LIA 46	GE	GENÈVE Saint-Antoine	1997/1998	1	LT B1-C1	Irregular	(Haldimann and Moinat 1999; Simon and Desideri 1999)
LIA 47	GE	MEYRIN Veyrot	1922	1	LT B2	Regular	(Kaenel 1990)
LIA 48	GR	CASTANEDA -	1970s	1	LT A/C	Regular	(Nagy 2000; Nagy 2008)
LIA 49	GR	TRUN Darvella	1963–1968	8	LT B2-C2	Regular	(Tanner 1980)
LIA 50	JU	CHEVENEZ Au Breuille	2012	1	LT	Regular	(Jory 2012; Moghaddam 2016)
LIA 51	LU	HOCHDORF -	1887	1	LT B2	Regular	(Moghaddam et al. 2013a, b)
LIA 52	LU	STANS Pfarrkirche	1984/1985	1	LT C2	Regular	(Lehner 1986)
LIA 53	LU	SURSEE Hofstetterfeld	2011	1	LT B2	Regular	(Nielsen 2014)
LIA 54	LU	SURSEE Moosgasse	1920s	1	LT	Regular	(Moghaddam 2016; Moghaddam et al. 2016; Moghaddam et al. 2018; Nielsen 2014)
LIA 55	NE	CORNAUX Les Sauges	1965/1966	21	LT D1	Irregular	(Ramseyer 2009; Schwab 1989)
LIA 56	NE	LA TÈNE (Collection Schwab)	1863–1911	16	LT	Irregular	(Alt and Jud 2007, 2009, 2013; Jud 2007)
LIA 57	NE	LE LANDERON Les Bévières	1990	1	LT A	Regular	(Hofmann and Simon 1991)
LIA 58	VD	AVENCHES Avenue Jomini	1992	2	HA/LT	Irregular	(Moinat 1993)
LIA 59	VD	LAUSANNE-VIDY Route de Chavannes	1975, 1989–1990	18	LT D1	Regular	(Debard 2014; Kaenel 1990, 1995; Müller et al. 1999)
LIA 60	VD	OLLON/ST. TRIPHON Le Lessus	1959, 1979	2	LT D	Regular	(Kaenel 1990)
LIA 61	VD	RANCES Vy des Buissons	1957	1	LT B1	Regular	(Kaenel 1990)
LIA 62	VS	BRAMOIS Panoë, Villa Lathion-Lopes & Schaller	1994–2008	17	LT B2-D2	Regular	(Curdy et al. 2009; Debard 2014; Moghaddam 2016; Moghaddam et al. 2016; Moghaddam et al. 2018)
LIA 63	VS	RANDOGNE Bluche	2001–2005	13	LT C1-D1	Regular	(Hofstetter 2018)
LIA 64	VS	SION Ancienne Placette	1992	1	LT D	Regular	(Curdy et al. 2009)
LIA 65	VS	SION Crypte de la Cathédrale	1988	1	LT D1	Regular	(Curdy et al. 2009)
LIA 66	VS	SION Nouvelle Placette	1986–1987	5	LT C2-D2	Regular	(Curdy et al. 2009)
LIA 67	VS	SION Parking Remparts	2006	11	LT C1-D2	Regular	(Debard et al. 2014)
LIA 68	VS	SION Petit Chasseur	1965–1992	8	LT B1-D1	Regular	(Curdy et al. 2009)
LIA 69	VS	SION Sous-le-Scex	1994	27	LT C1-D2	Regular	(Curdy et al. 2009; Lehner 1987)
LIA 70	VS	SION Passage de La Matze	1998	2	LT D	Regular	(Julie Debard 2014; Moret et al. 2000)
LIA 71	ZG	ZUG Oberwil	1951	1	LT B?	Regular	(Bauer 1996)
LIA 72	ZH	ANDELFINGEN Hochlaufen	1911	16	LT B	Regular	(Challet 1997; Viollier 1912)
LIA 73	ZH	FÄLLANDEN Fröschbach	1992	1	LT C	Regular	(Fischer 1994)
LIA 74	ZH	WETZIKON Sandbüel	1911	1	LT C1	Regular	(Altorfer and Schmid 1996)
LIA 75	ZH	ZÜRICH Kernstrasse	1903, 2017	2	LT C1-C2	Regular	(Bucher et al. 2019)

The variables considered in our review include representation/preservation of human remains, types of funerary treatment, demographic patterns (sex, age at death), paleopathology (dental caries, cribra orbitalia, trauma), and data from stable isotope ratio studies.

When discussing demographic patterns, we followed the age class subdivision commonly adopted by Swiss anthropological reports: infans I (0–7 years old), infans II (7–14 years old), juvenile (14–20 years old), adult (40–60 years old), mature (40–60 years old), senile (≥ 60 years old).

Our choice of focusing on cribra orbitalia, dental caries, and trauma was dictated by the amount and quality of available data. Little paleopathological research has been conducted using skeletal material from the La Tène period in Switzerland (Curdy et al. 2009; Gallay 2014; Laffranchi et al. 2021; Moghaddam et al. 2013a, b; Moghaddam et al. 2015; Ramseier et al. 2005). In this regard, systematic and epidemiological studies are especially rare.

Cribra orbitalia is thought to be associated with iron deficiency anemia. Various factors can contribute to the formation of Cribra orbitalia, particularly not only parasitic infestation and infectious diseases and diarrheal diseases but also malnutrition or changes in diet (Hengen 1971; Mensforth et al. 1978; Reinhard 1992; Stuart-Macadam 1982, 1987a, 1987b; Stuart-Macadam 1989a, b; Stuart-Macadam 1989a, b; Stuart-Macadam and Kent 1992; Wadsworth 1992; Weinberg 1992). Vitamin C deficiency (Grupe 1995), vitamin B12 or folic acid deficiency (Walker et al. 2009), inflammation, and hemorrhagic processes (Carli-Thiele and Schultz 1999) have also been discussed as causes of Cribra orbitalia.

Carbohydrates (sugars and starches) play a major role in the development of dental caries. Plaque pH falls within minutes after administration of sugar, and, to a lesser degree, starches. Frequent sugar consumption and foods with both sugars and starches are highly cariogenic (Hillson 1996). An examination of dental caries can therefore be used to assess dietary patterns.

For the study of postcranial trauma, we compiled a dataset from the regular burial sites for which paleopathological data was published in sufficient detail and which contained complete skeletons rather than only skulls. We added the specimens of Münsingen-Rain to the aforementioned subsample for the study of cranial trauma.

Stable isotope ratios of carbon and nitrogen (δ^13^C, δ^15^N) are often used in archeology for reconstructing past dietary patterns (e.g., dietary contribution of C_3_ vs. C_4_ plants, access to animal proteins, exploitation of terrestrial vs. marine vs. freshwater resources) (Katzenberg 2007; Schoeninger 2011). Isotopic ratios of oxygen and strontium (δ^18^O, ^87^Sr/^86^Sr), and, less frequently, sulfur (δ^34^S) are usually explored for detecting patterns of regional nonlocality and mobility (Lightfoot and O'Connell 2016; Montgomery 2010). In this work, we include an overview of this type of studies for the Swiss territory, in order to highlight the possible presence of main tendencies for this geographic and chronological context.

For some sites, anthropological data are listed in unpublished or preliminary reports (Dietrich et al. 2007, 2009a, b; Maroelli and Gallay 2014; Moinat 2009). In other instances, only scarce information is available due to the extremely poor preservation of the skeletal remains (Costa 2005; Del Fattore 2007; Kaenel 1990; Moghaddam 2016). We decided not to include these cases in our summary statistics but rather to consider them only when discussing our data. For the same reason, we did not attempt an exploration of fine-grained chronological or regional patterns in our dataset, but focused on single archeological and anthropological variables and explored these using all contexts and individuals for which the relevant data was available.

Finally, in order to provide a broader contextualization to our results, we collected a comparative dataset based on the same criteria followed for the Iron Age including 71 funerary contexts from the Swiss territory and chronologically spanning from the Neolithic (ca. 6500–2200 BCE) to the post-medieval period (ca. 16^th^–19^th^ century CE) (Table 3).

**Table 3:** Reference data from Switzerland that were used in this study

Dating	Site code	Canton	Site name	References
Neolithic	NEO 01	AG	SPREITENBACH Moosweg	(Bleuer et al. 1999, 2012 Meyer and Alt 2012)
	NEO 02	AG	LENZBURG Goffersberg	(Scheffrahn 1967; Scheffrahn 1998)
	NEO 03	AG	ZURZACH Schlosspark/Himmelreich	(Doswald et al. 1989)
	NEO 04	BE	NIEDERRIED Ursisbalm	(Hug 1956)
	NEO 05	SH	SCHAFFHAUSEN Schweizersbild	(Scheffrahn 1967)
	NEO 06	VS	PULLY Chamblandes	(Moinat and Simon 1986)
	NEO 07	VS	COLLOMBEY-MURAZ Barmaz I/II	(Simon and Kramar 1986)
Bronze Age	BRA 01	BE	LAKE THUN REGION Several locations	(Ulrich-Bochsler and Cooper 2010)
	BRA 02	BS	BASEL Riehen/Britzigerwald	(Fischer 1997)
	BRA 03	FR	MURTEN Löwenberg	(Fischer 1997)
	BRA 04	FR	POSIEUX Bois de Châtillon	(Simon and Kaufmann 1998)
	BRA 05	GR	DOMAT/EMS Crestas, Parzelle 535	(Seifert 2000)
	BRA 06	VD	ORBE Boscéax	(Simon and Kaufmann 1998)
	BRA 07	VD	VUFFLENS-LA-VILLE En Sency	(Simon and Kaufmann 1998)
	BRA 08	VS	GRIMISUAT Champlan	(Simon and Kaufmann 1998)
	BRA 09	VS	SION Petit Chasseur	(Simon and Kaufmann 1998)
	BRA 10	VS	COLLOMBEY-MURAZ Barmaz I	(Simon and Kaufmann 1998)
	BRA 11	ZH	FÄLLANDEN Fröschbach	(Fischer 1997)
	BRA 12	ZH	RAFZ Badener-Landstrasse	(Fischer 1997; Graf 1993)
	BRA 13	ZH	WALLISELLEN Föhrlibuck	(Fischer 1997)
Early Iron Age	EIA 01	AG	WOHLEN -	(Tatarinoff 1925)
	EIA 02	AG	LENZBURG Lindwald	(Drack 1992)
	EIA 03	BE	ATTISWIL Wybrunne	(Ulrich-Bochsler 2010)
	EIA 04	FR	RIED Mühlehölzli	(Kaufmann and Schoch 1983)
	EIA 05	SH	HEMISHOFEN Sankert	(Drack 1992)
	EIA 06	SO	OBERGÖSGEN Oberhard	(Drack 1992)
	EIA 07	TI	DALPE -	(Primas 1970)
	EIA 08	TI	MESOCCO Coop	(Martelli 2002)
	EIA 09	ZH	KLOTEN Homberg	(Drack 1980)
Antiquity/Roman	ROM 01	AG	KAISERAUGST Höll	(Brunner 2014)
	ROM 02	AG	KAISERAUGST Rheinstrasse/Stalden	(Bay 1968)
	ROM 03	AG	WINDISCH Rebengässli	(Schoch et al. 1989)
	ROM 04	BE	WORB Worbberg	(Ramstein and Ulrich-Bochsler 2006)
	ROM 05	BE	STUDEN Wydenpark	(Lösch et al. 2013)
	ROM 07	FR	TAFERS Windhalta	(Kaufmann and Schoch 1990)
	ROM 08	JU	COURROUX Derrière la Forge	(Martin-Kilcher 1976)
Early Medieval	EMA 01	BE	KÖNIZ Buchsi	(Ulrich-Bochsler and Meyer 1990a, 1990b)
	EMA 02	BE	KALLNACH Bergweg	(Ulrich-Bochsler 2006)
	EMA 03	BE	SEEBERG Pfarrkirche	(Heigold-Stadelmann and Ulrich-Bochsler 2009)
	EMA 04	BE	OBERWIL BEI BÜREN Reformierte Pfarrkirche	(Ulrich-Bochsler et al. 1985)
	EMA 05	BE	BÜETIGEN Hauptstrasse	(Ulrich-Bochsler 1994)
	EMA 06	BE	BERN Bümpliz, Mauritiuskirche/Bienzgut	(Cooper et al. 2017)
	EMA 07	BE	KÖNIZ-NIEDERWANGEN Sonnhalde	(Ulrich-Bochsler et al. 2008)
	EMA 08	BE	UNTERSEEN Obere Gasse 42	(Ulrich-Bochsler 1994)
	EMA 09	LU	AESCH Zielacher	(Cueni 2009)
	EMA 10	FR	RIED Mühlehölzli	(Kaufmann and Schoch 1983)
	EMA 11	GR	BONADUZ Valbeuna	(Brunner 1972)
	EMA 12	JU	COURROUX Place des Mouleurs	(Cooper et al. 2016a, b)
	EMA 13	TG	GÜTTINGEN Grauer Stein	(Kaufmann et al. 1989)
	EMA 14	ZG	BAAR Zugerstrasse	(Horisberger et al. 2004)
	EMA 15	ZH	ELGG Ettenbühl	(Langenegger 1995)
High/Late Medieval	HLM 01	BE	ROHRBACH Reformierte Pfarrkirche	(Ulrich-Bochsler 1988a, b)
	HLM 02	BE	WALKRINGEN Kirche	(Ulrich-Bochsler and Meyer 1992)
	HLM 03	BE	STEFFISBURG Reformierte Kirche	(Ulrich-Bochsler and Meyer 1994)
	HLM 04	BE	LEUZIGEN Kirche	(Ulrich-Bochsler 1989)
	HLM 05	BE	WANGEN AN DER AARE Reformierte Pfarrkirche	(Ulrich-Bochsler and Schäublin 1991)
	HLM 06	BE	TWANN Pfarrkirche	(Ulrich-Bochsler 1988a, b)
	HLM 07	BE	TWANN St. Petersinsel	(Ulrich-Bochsler 1987)
	HLM 08	BE	ZWEISIMMEN Kirchgasse (Group A)	(Somers et al. 2017)
	HLM 09	BE	BÜREN AN DER AARE Oberbüren, Chilchmatt	(Christen and Cuendet 2006)
	HLM 10	BE	SEEBERG Pfarrkirche (Groups 1–4)	(Heigold-Stadelmann and Ulrich-Bochsler 2009)
	HLM 11	BE	UNTERSEEN Kirche	(Ulrich-Bochsler et al. 2001**, **2008)
	HLM 12	BE	MADISWIL Kirche	(Ulrich-Bochsler et al. 2008)
	HLM 13	GR	TOMILS Sogn Murezi	(Papageorgopoulou 2008)
	HLM 14	SZ	SCHWYZ Pfarrkirche St. Martin (Group 1–2)	(Cueni 1995)
Post-medieval	POM 01	BE	LAUENEN Reformierte Pfarrkirche	(Ulrich-Bochsler 1990)
	POM 02	BE	AEGERTEN Kirche Bürglen (groups 2–5)	(Ulrich-Bochsler 1990)
	POM 03	BE	BERN Holzwerkhof &Sidlerstrasse	(Ulrich-Bochsler et al. 2016)
	POM 04	BE	WANGEN AN DER AARE Reformierte Pfarrkirche (Group 3, 4, 7)	(Ulrich-Bochsler and Schäublin 1991)
	POM 05	BE	ZWEISIMMEN Kirchgasse (Group B)	(Somers et al. 2017)
	POM 06	SZ	SCHWYZ Pfarrkirche St. Martin (Group 3–5)	(Cueni 1995)

A word for the wise: we are fully aware of the pitfalls associated with grouping data of varying quantity and quality, contexts from a fairly large region and timespan as well as from different types of archeological settings in the same study. However, we also believe that the results from such an approach, if considered critically, have the potential to shed some light on a larger picture (especially regarding potential demographic and broad chronological aspects) otherwise “buried” in the often difficult to find specialist reports and/or case studies (cf. Alterauge et al. 2020; Milella et al. 2015; Moghaddam and Lösch 2015).

This, in conjunction with the bibliography collected by our study, may provide a useful—although necessarily rough—basis for better defining open research questions to address by further, more detailed analyses.

## Results

### Representation of the remains

In the selected sample of sites, roughly three-quarters of the skeletons which were originally present were not preserved or not stored (Table 4).

**Table 4:** Proportion of graves with preserved/collected human remains at different sites

Site	*N* originally present graves	*N* with preserved/collected human remains	% with preserved/collected human remains
LIA 34	40	8	20.0
LIA 28	220	77	35.0
LIA 72	29	16	55.2
LIA 54	4	1	25.0
LIA 49	27	8	29.6
LIA 56	50–100	16	16.0–32.0
LIA 48	90	1	1.1
Total	460–510	127	24.9–27.6

The true proportion is most likely even much higher because only sites with preserved human remains and published anthropological results were considered in this study. For example, Kaenel (1990) listed a total of 232 grave inventories from Western Switzerland, but anthropological data were available for only 8 of them (3.4%). Altogether, we assume that bones were preserved and/or collected from less than 10% of all excavated graves.

### Funerary rites: cemeteries and isolated graves

In the Early Iron Age (Hallstatt period), the dead were buried inside tumuli. Most were cremated, but during this period a shift took place and inhumations appeared alongside cremations. In Western Switzerland, the new custom began earlier than in Eastern and Southern Switzerland, where cremations continued for longer. At the beginning of the La Tène period, the custom of cremating the dead was completely replaced by inhumations in a gradual process (Lüscher and Müller 1999). In LT A, several inhumations still took place in older tumuli from the Hallstatt period, for example in Orny-sous-Mormont (Maroelli and Gallay 2014) or Murten-Löwenberg (Kaenel 1990), but generally the La Tène period is characterized by inhumations in flat graves (Kaenel 1990; Lüscher and Müller 1999).

Different types of grave constructions have been identified, including grave chambers or rows of stones, and stones covering graves that are thought to have originally been placed on a coffin lid. Coffins were made from boards or hollowed tree trunks. Female graves often contain sets of jewelry and fibulas, whereas men were sometimes interred with arms such as swords and lances (Lüscher and Müller 1999). Another shift occurred towards the end of the Late Iron Age, when the cremation of the dead reappeared around 150 BCE and consequently became established again (Müller and Lüscher 2004; Ruffieux et al. 2006; Suter et al. 1990).

**Fig. 2:**
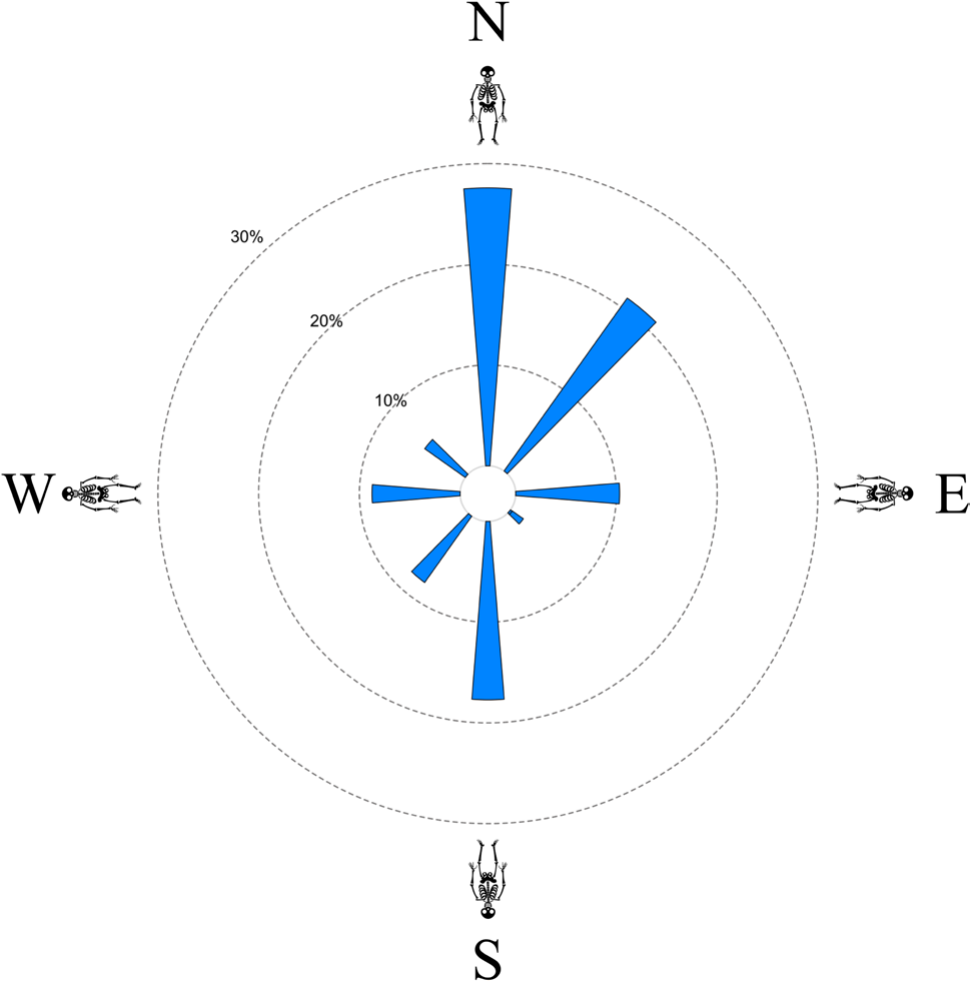
Frequencies of different orientations in Late Iron Age burials in Switzerland (*N* = 446)

The orientation has been reported for 34 sites and 446 individuals in our sample (Fig. 2, Table 5).

**Table 5:** Orientation of Late Iron Age burials in Switzerland (regular burials)

	N-S	S–N	E-W	W-E	NE-SW	SE-NW	NW–SE	SW-NE	TOTAL
LIA 01	0	1	2	0	0	1	1	0	5
LIA 09	11	21	0	0	0	0	0	0	32
LIA 12	1	0	0	0	0	0	0	0	1
LIA 19	0	0	0	1	0	0	0	0	1
LIA 21	4	1	0	0	0	0	0	0	5
LIA 28	76	28	38	36	8	15	17	3	221
LIA 34	4	1	0	1	0	0	0	0	6
LIA 35	0	0	0	0	0	1	0	0	1
LIA 36	1	0	0	0	0	0	0	0	1
LIA 39	1	0	0	0	0	0	0	0	1
LIA 41	0	1	0	0	0	0	0	0	1
LIA 42	0	0	0	0	5	0	0	0	5
LIA 43	1	5	0	0	2	0	3	2	13
LIA 44	0	1	0	0	5	0	0	0	6
LIA 49	0	0	6	0	0	2	0	0	8
LIA 51	1	0	0	0	0	0	0	0	1
LIA 52	0	0	0	0	1	0	0	0	1
LIA 53	0	1	0	0	0	0	0	0	1
LIA 57	0	0	0	1	0	0	0	0	1
LIA 59	8	10	0	0	0	0	0	0	18
LIA 60	1	0	0	0	0	0	0	1	2
LIA 62	0	0	0	0	15	0	0	0	15
LIA 63	0	0	0	0	13	0	0	0	13
LIA 64	1	0	0	0	0	0	0	0	1
LIA 65	0	0	0	0	1	0	0	0	1
LIA 66	0	0	0	0	5	0	0	0	5
LIA 67	11	0	0	0	0	0	0	0	11
LIA 68	0	0	0	0	7	0	0	1	8
LIA 69	0	0	0	0	28	0	0	0	28
LIA 70	0	0	0	0	2	0	0	0	2
LIA 72	0	8	0	0	1	17	1	0	27
LIA 73	0	1	0	0	0	0	0	0	1
LIA 74	0	0	0	0	1	0	0	0	1
LIA 75	2	0	0	0	0	0	0	0	2
Total	123	79	46	39	94	36	22	7	446

These include all burials from Münsingen-Rain (Hodson 1968), even though human remains are only preserved from a fraction of these graves. For the other sites, we considered only the orientations of those individuals for which anthropological data exists as well. The graves were predominantly oriented on the N-S (head in the north, feet in the south) or S–N axis. Less than a quarter of all graves were oriented towards the East or the West. Altogether, more than half of the graves faced the south, southwest, or southeast (53%), while another 28% faced the north, northeast, or northwest. Only 19% were oriented towards the east or the west.

### Funerary rites: “irregular” burials

A number of sites yielded unusual burials, including complete and partial skeletons in pits in settlements and sanctuaries, skeletons in a seated/hyperflexed position, and human remains in rivers and lakes. For the sake of simplicity, we grouped them together even though their interpretations may vary.

During an excavation from 2006 to 2009 on the hill of La Sarraz-Le Mormont, an alleged Celtic sanctuary, a surface of more than 8000 m^2^ with about 260 pits was excavated. The pits contained depositions of offerings which comprised various metal objects, jewellery, millstones, ceramics, tools, coins, and animals. Human remains were also found in the pits (Dietrich et al. 2007, 2009a, b). They comprised complete skeletons as well as isolated bones and body parts. Some of the complete skeletons were found extended supine or prone, others were hyperflexed, and in some cases, the position of the limbs suggested a sitting posture. Some individuals appeared to have been carelessly thrown into the pits. Traces of various types of manipulation were identified on the bones. These were consistent with a large range of actions including exposition to fire, and severing of heads and other body parts as well as evisceration (Moinat 2009).

Individuals buried in a seated or hyperflexed position are also known from Avenches and Geneva (Bonnet et al. 1989; Moinat 1993). The interpretation of these burials is uncertain, but a cultic background is suspected.

The remains of 50–100 individuals were recovered from the La Thielle River in La Tène until the completion of the excavations in 1917. Most bones have since been lost. Some of the preserved long bones were gnawed by carnivores, indicating that the individuals had been at the surface for some time before being deposited in the river (Alt and Jud 2009). The circumstances of the death and the deposition of these individuals in the river are disputed. The abundance of arms at the site suggested a battle background and the human remains have been interpreted as trophies that were attached to the bridge (Müller 2009). However, the demographic composition with children, women, and men, the absence of typical war-related injuries, and instead the presence of suspected traces of post-mortem manipulations, may rather be indicative of human sacrifices, though it cannot be excluded that the individuals were captured non-combatants (Alt et al. 2007). It has also been stated that these findings are to be seen in connection with manipulations of the deceased that are increasingly known from funerary contexts as well and could be considered as exceptional funerary practices rather than human sacrifices (Alt and Jud 2009; Jud 2007).

In close proximity to La Tène, more human remains were discovered in a river bed in proximity to the Iron Age bridge of Cornaux-Les Sauges. The complete skeletons were found in random positions, and in some cases among and under pales from the original bridge. Originally, this site was thought to be a sanctuary (Müller et al. 1999; Müller and Lüscher 2004; Wyss et al. 2002). After a re-examination, Ramseyer (2009) interpreted the findings to be more consistent with the collapse of the bridge, possibly as a result of a large group of people crossing it.

Commingled remains, especially skulls, of at least 21 individuals were recovered from the Celtic port in Geneva. They comprised not only mainly young adults, both male and female, but also children and have been interpreted by Bonnet et al. (1989) as human sacrifices on the basis of the demographic composition, numerous peri-mortem traumas, and/or manipulations and the fact that they did not appear to have been deposited simultaneously but over a longer period of time. These data contradict the fact that they were victims of a single massacre or battle.

The two necropolises of the unfortified proto-urban settlement in Basel-Gasfabrik site yielded a total of almost 200 individuals, but additional skeletal remains representing a minimum number of 130 individuals have been found within the settlement. These comprise 28 complete skeletons from pits and wells and isolated bones from various contexts. Unlike the skeletons in the cemeteries, about 15% of the isolated bones from the settlement showed signs of various peri- and postmortem manipulations that included signs of scorching, as well as various sharp force traumas. Bones from pits in the settlement have been interpreted as relicts of multi-level funerary rites (Pichler et al. 2013).

### Demographic patterns

Information about age-at-death and sex are available for 478 individuals from 75 sites. Of these, 363 (75.9%) are adults and 115 (24.1%) are nonadults. The adults include 143 females (39.4%), 164 males (45.2%), and 56 individuals of undetermined sex (15.4%). The nonadults include 67 infans I (of which 10 are neonates), 23 infans II, and 17 juveniles (Table 6).

**Table 6:** Demographic composition of the skeletal assemblages from Late Iron Age sites in Switzerland

Site	Adults			Children			
	Female	Male	Undetermined	Infans I	Infans II	Juvenile	Unspecified
LIA 01	3	4	1				
LIA 02	1						
LIA 03	1			1			
LIA 04	1						
LIA 05	2					1	
LIA 06		1					
LIA 07	4						
LIA 08	1	1					
LIA 09	11	3	7	13	5		
LIA 10	1						
LIA 11	2	2					
LIA 12	1						
LIA 13	1						
LIA 14	1						
LIA 15	2	2					
LIA 16	1						
LIA 17		1					
LIA 18		1					
LIA 19	1						
LIA 20	2	1					
LIA 21	1			3		1	
LIA 22	1			1			
LIA 23	1						
LIA 24	1						
LIA 25						1	
LIA 26		1					
LIA 27	1						
LIA 28	32	34		1	6	4	
LIA 29	1	1					
LIA 30	1	2	2	1			
LIA 31		1					
LIA 32	1	1					
LIA 33	1	1					
LIA 34	4	2		1		1	
LIA 35	1						
LIA 36	1						
LIA 37	2	1					
LIA 38	1						
LIA 39	1						
LIA 40	3	5	7	24	3	1	
LIA 41	1						
LIA 42	1		2	1	1	1	
LIA 43	5	7	3	5		1	
LIA 44	2	1		3		1	
LIA 45			17				4
LIA 46		1					
LIA 47	1						
LIA 48						1	
LIA 49	2	6					
LIA 50				1			
LIA 51	1						
LIA 52				1			
LIA 53	1						
LIA 54		1					
LIA 55		14	5		1	1	
LIA 56	3	8	1		2	1	1
LIA 57	1						
LIA 58		1	1				
LIA 59	5	3		9		1	
LIA 60				2			
LIA 61		1					
LIA 62	5	11	1				
LIA 63		13					
LIA 64		1					
LIA 65				1			
LIA 66	1	4					
LIA 67	1	5	4		1		
LIA 68	2	3			1	1	1
LIA 69	13	9	3		2		
LIA 70	1		1				
LIA 71	1						
LIA 72	6	8	1			1	
LIA 73					1		
LIA 74		1					
LIA 75	1	1					
Total La Têne	143	164	56	68	23	18	6

**Fig. 3:**
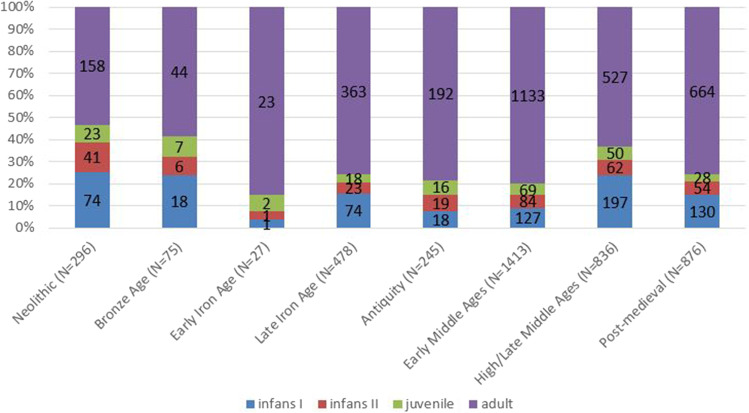
Demographic composition of skeletal samples from the Neolithic to the post-medieval period in Switzerland. The sample from the Antiquity/Roman period does not include skeletons from settlements

**Fig. 4:**
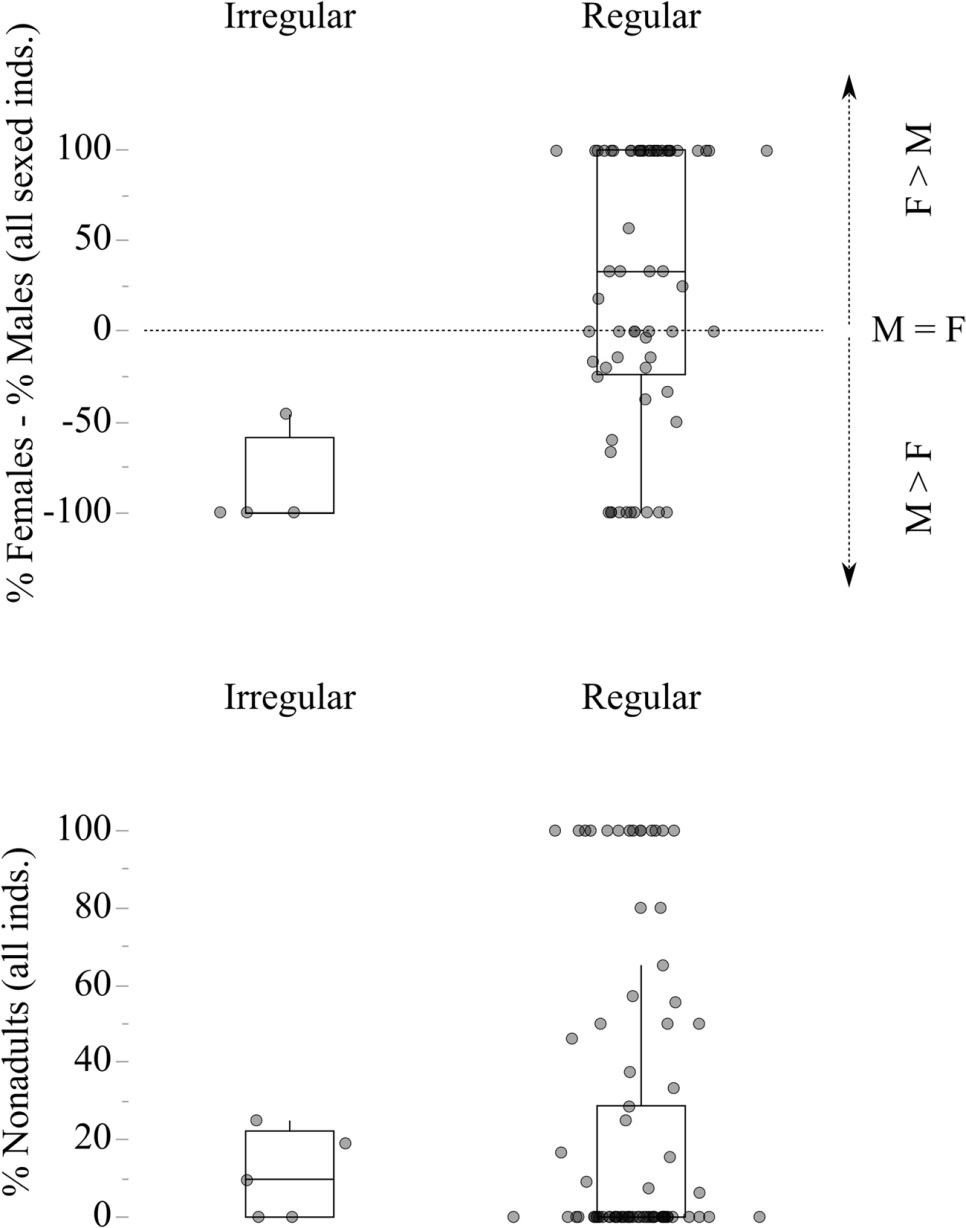
Frequency of sexes and nonadults in irregular and regular Late Iron Age contexts. Each dot represents a funerary context. The frequency of males and females is calculated relative to the number of sexed individuals

To better contextualize the demographic composition of the La Tène sample, we compare it with summary statistics from other time periods (Table 7, Fig. 3) and furthermore differentiate between regular and irregular contexts (Fig. 4).

**Table 7:** Demographic composition of the reference sites from Switzerland

Site	*N* adult	*N* infans I	*N* infans II	*N* juvenile
NEO 01	9	2	1	1
NEO 02	38	13	16	9
NEO 03	2	0	0	0
NEO 04	1	0	0	2
NEO 05	5	15	2	0
NEO 06	67	30	11	8
NEO 07	36	14	11	3
Total Neolithic	158	74	41	23
BRA 01	7	2	3	1
BRA 02	1	0	0	0
BRA 03	0	0	0	1
BRA 04	1	0	0	0
BRA 05	1	0	0	0
BRA 06	0	2	0	1
BRA 07	7	0	0	2
BRA 08	5	1	0	0
BRA 09	3	5	1	1
BRA 10	14	2	0	0
BRA 11	3	6	2	1
BRA 12	1	0	0	0
BRA 13	1	0	0	0
Total Bronze Age	44	18	6	7
EIA 01	1	0	0	0
EIA 02	1	0	0	0
EIA 03	1	0	0	0
EIA 04	2	1	0	0
EIA 05	2	0	0	0
EIA 06	1	0	0	0
EIA 07	6	0	0	1
EIA 08	5	0	0	1
EIA 09	4	0	1	0
Total Early Iron Age	23	1	1	2
ROM 01	35	1	1	3
ROM 02	48	7	10	4
ROM 03	23	2	3	2
ROM 04	1	0	0	0
ROM 05	3	1	0	0
ROM 07	53	4	4	7
ROM 08	29	3	1	0
Total Roman	192	18	19	19
EMA 01	35	2	2	0
EMA 02	117	20	7	8
EMA 03	22	19	0	1
EMA 04	50	4	3	5
EMA 05	17	0	0	0
EMA 06	9	1	0	0
EMA 08	7	1	2	0
EMA 09	50	6	4	1
EMA 10	72	11	13	3
EMA 11	498	29	39	39
EMA 12	39	14	1	0
EMA 13	41	3	5	0
EMA 14	18	3	1	3
EMA 15	158	14	7	9
Total Early Medieval	1133	127	84	69
HLM 01	27	19	6	2
HLM 02	30	15	5	3
HLM 03	72	32	4	6
HLM 04	2	10	0	0
HLM 05	5	2	0	0
HLM 06	11	9	4	0
HLM 07	27	1	4	1
HLM 08	50	4	6	5
HLM 13	167	94	21	18
HLM 14	136	11	12	15
Total High/Late Medieval	527	197	62	50
POM 01	1	5	0	0
POM 02	20	20	5	2
POM 03	384	27	15	16
POM 04	20	44	4	0
POM 05	45	22	2	0
POM 06	194	12	28	10
Total Post-Medieval	664	130	54	28

### Paleopathology

#### Cribra orbitalia

The prevalence of Cribra orbitalia has been reported for sites in the cantons of Bern and Luzern (Moghaddam 2016), Basel (Pichler et al. 2015), and Vaud and Valais (Debard 2014) (Table 8).

**Table 8:** Prevalence of Cribra orbitalia in the La Tène and reference samples (five additional skulls from Münsingen-Rain (LIA 28) that had previously been missing were examined by the authors and added to the sample reported by Moghaddam (2016))

Site	Adults			Children			Total		
	*N*	*n*	%	*N*	*n*	%	*N*	*n*	%
LIA 09	39	8	20.5	13	3	23.1	52	11	21.2
LIA 28	65	7	10.8	12	1	8.3	77	8	10.4
LIA 21	2	0	0	0	0	0	2	0	0
LIA 03, 04, 05, 06, 07	12	1	8	2	0	0	14	1	7.1
LIA 34	9	0	0	2	2	100	11	2	18.2
LIA 30	1	1	100	0	0	0	1	1	100
LIA 01	6	1	16.7	0	0	0	6	1	16.7
LIA 51, 52, 53	3	0	0	1	1	100	4	1	25.0
Total La Tène (BE/LU)	137	18	13.1	30	7	23.3	167	25	15.0
LIA 40	0	0	0	45	6	13.3	45	6	13.3
LIA 59	7	1	14.3	11	0	0	18	1	5.6
LIA 62	17	0	0	0	0	0	17	0	0
LIA 66	5	0	0	0	0	0	5	0	0
LIA 68	5	0	0	3	0	0	8	0	0
LIA 67	10	0	0	1	0	0	11	0	0
LIA 69	25	0	0	3	0	0	28	0	0
Total La Tène	206	19	9.2	93	13	14.1	299	32	10.7
EMA 12	36	1	2.8	10	6	60	46	7	15.2
EMA 07	10	1	10	12	3	25	22	4	18.2
EMA 02	77	15	19.5	18	9	50	95	28	29.5
Total Early Medieval	123	17	13.8	40	18	45.0	163	39	23.9
HLM 12	0	0	0	16	3	18.8	16	3	18.8
HLM 11	31	2	6.5	9	3	33.3	40	5	12.5
HLM 09	120	10	8.3	26	9	34.6	148	19	12.8
HLM 08	14	2	14.3	4	2	50	18	4	22.2
Total High/Late Medieval	165	14	8.5	55	17	30.9	222	31	14.0
POM 05	37	3	8.1	8	3	37.5	45	6	13.3
POM 03	178	28	15.7	25	8	32	203	36	17.7
Total Post-Medieval	215	31	14.4	33	11	33.3	248	42	16.9

**Fig. 5:**
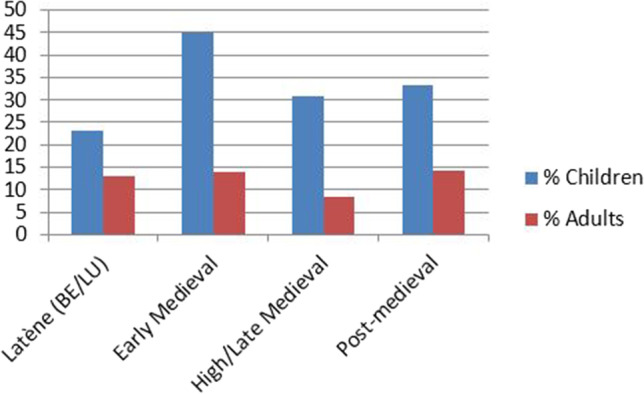
Prevalence of Cribra orbitalia in children and adults in different periods (only the data from the cantons of Bern and Luzern is considered here)

For the following considerations, only the data from the cantons of Bern and Luzern (Moghaddam 2016) are used because it could be ascertained that they were recorded in a manner consistent with the reference groups in that only individuals with at least one preserved orbit were considered. Compared to the medieval and post-medieval samples, the prevalence of Cribra orbitalia in the Late Iron Age is low in children, whereas the adult prevalence does not stand out (Fig. 5). However, the sample size for children is very small and even the seemingly large difference between Late Iron Age and early medieval children is not statistically significant (Fisher’s exact test *P* = 0.0797). Suitable reference data from other prehistoric periods in Switzerland is not available to date.

#### Dental caries

Caries prevalence by tooth has been calculated for the sites from the canton of Bern (Moghaddam 2016). In adults, 79/1468 teeth were found to exhibit carious lesions and the total prevalence is therefore 5.4%. We chose the reference groups only from this canton as well for the purpose of methodological consistency, and to avoid a bias due to possible regional differences (Table 9). In comparison to sites from later periods, the caries prevalence is very low in the Late Iron Age. It is consistent with the values from the Bronze Age and Antiquity, but only one individual represents the latter. Altogether, the prehistoric groups appear to have a similar prevalence of caries. A sharp increase only occurs during the Middle Ages.

**Table 9:** Caries prevalence by tooth in adults from the canton of Bern (three additional skulls with teeth from Münsingen-Rain (LIA 28) that had previously been missing were examined by the authors and added to the sample reported by Moghaddam (2016))

Period	Site	*N* teeth	*N* carious	% carious
La Tène	LIA 01	85	1	1.2
	LIA 02–07	92	3	3.3
	LIA 09	437	29	6.6
	LIA 21	27	7	25.9
	LIA 28	767	36	4.7
	LIA 30	19	2	10.5
	LIA 34	41	1	2.4
	Total	1379	78	5.7
Bronze Age	BRA 01	121	10	8.3
	Total	121	10	8.3
Antiquity	ROM 04	28	2	7.1
	Total	28	2	7.1
Early Middle Ages	EMA 01	254	51	20.1
	EMA 02	1151	210	26.4
	EMA 05	89	24	27
	EMA 06	104	12	11.5
	Total	1598	297	18.6
High/Late Middle Ages	HLM 09	2575	619	24
	HLM 01	328	49	14.9
	HLM 10	251	40	21.6
	HLM 08	416	120	28.8
	Total	3570	828	23.2
Post-medieval	POM 05	549	271	49.4
	POM 03	2179	1136	52.1
	Total	2728	1407	51.6

#### Trauma

##### Regular burials

Fractures were reported in seven adult individuals, three of which were female. In four cases, a long bone was affected (2 clavicles, 1 forearm, 1 tibia). The remaining cases were fractures of hand or foot bones and ribs. All postcranial traumata were healed, and all are consistent with an accidental cause (Galloway 2014a, b).

Cranial trauma was identified in 9 out of 231 individuals (1 female and 8 males, all adult) (Table 10). The traumata affected the mandible in three cases and the cranial vault in the remaining six cases. A male from Kerzers exhibited a lethal injury to the back of his head (Ramseyer 1997) but all other traumata were healed. Because no details about the types of traumata were published, we cannot make a statement about whether they more likely resulted from interpersonal violence or accidents.

**Table 10:** Trauma in Late Iron Age regular burial sites in Switzerland (five additional skulls from Münsingen-Rain (LIA 28) that had previously been missing were examined by the authors and added to the sample reported by Moghaddam (2016))

Site	Postcranial trauma				Cranial trauma			
	*N*	*n*	%	Details	*N*	*n*	%	Details
LIA 01	8	0	0		8	0	0	
LIA 02–07	11	0	0		11	0	0	
LIA 09	39	0	0		39	1	2.6	Female: mandibular condyle, healed
LIA 21	5	1	20.0	Female: tibia	5	0	0	
LIA 28	-	-	-		76	5	6.6	Male: vault, healed
								Male: vault, healed
								Male: vault, healed
								Male: vault, healed
								Male: mandible, healed
LIA 30	5	0	0		5	0	0	
LIA 34	8	0	0		8	1	12.5	Male: vault, healed
LIA 44	7	0	0		7	1	14.3	Male: vault, peri-mortem
LIA 62	17	3	17.6	Male: clavicle, ribs	17	0	0	
				Female: clavicle				
				Male: carpal bone?				
LIA 64	1	1	100	Male: ulna/radius, metacarpal	1	0	0	
LIA 65	1	0	0		1	0	0	
LIA 66	5	0	0		5	0	0	
LIA 67	11	0	0		11	0	0	
LIA 68	8	0	0		8	0	0	
LIA 69	27	2	3.7	Female: metatarsal	27	1	3.7	Male: mandible
				Male: rib				
LIA 70	2	0	0		2	0	0	
Total	155	7	4.5		231	9	3.9	

#### Irregular burials

**Fig. 6:**
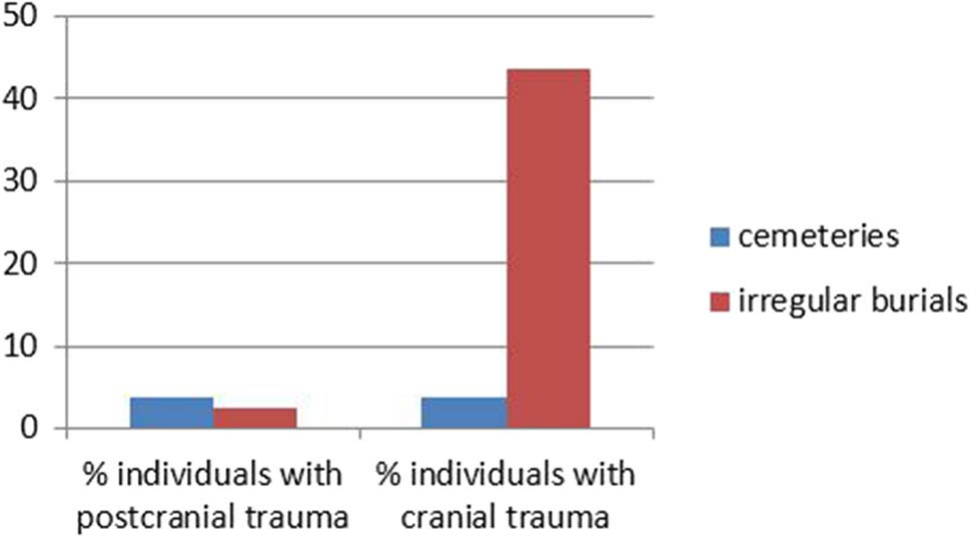
Percentage of individuals with cranial and postcranial trauma in Late Iron Age regular and irregular burial sites

Only one case of postcranial trauma was reported from irregular contexts (an individual from Avenches featuring a distal facture of the humerus with a pseudoarthrosis formed in the process of healing). All other traces of trauma at these sites were identified on cranial bones. They represent 39 individuals of which 17 (43.6%) showed traumatic lesions (Fig. 6, Table 11).

**Table 11:** Prevalence of cranial trauma in Late Iron Age irregular burials

Site	*N* crania	*N* affected	% affected
LIA 45	21	10	47.1
LIA 56	16	7	43.8
LIA 58	2	0	0

Two individuals with signs of trauma were recovered from a pit in the settlement at Basel-Gasfabrik. A young man had been buried prone with his feet cut off and deposited at the upper body. The second individual, also a male, was located partly under the first and exhibited an injury to the skull (Pichler et al. 2013).

The commingled remains from the Celtic port of Geneva comprise mainly cranial bones of not only young adults (males and females in similar proportions) but also subadults. Peri-mortem trauma was found on a large proportion of the cranial bones (10/21 individuals). The lesions were attributed to sharp force, suspected arrow injuries, fractured condyles and mastoids, and possible decapitations (Bonnet et al. 1989).

Out of 16 skulls from the river in La Tène, seven exhibited lesions, but the cause was only narrowed down in three cases. Individual 1, a male, was decapitated by several violent sharp blows that severed a part of the cranial base. It is suspected that this may have taken place post-mortem. Seven sharp force injuries were found on the skull of individual 5 (male). They are mainly located on the frontal bone and appear to have been inflicted from the same angle. This indicates that they were most likely delivered to an immobilized person and were therefore more likely inflicted post-mortem. The third individual (undetermined sex) had two severe blunt force injuries at the back of the skull, which were interpreted as the cause of death (Alt and Jud 2007, 2009; Alt et al. 2007).

Some skulls from the river at the site of a Celtic bridge in Cornaux-Les Sauges showed blunt force injuries which Ramseyer (2009) thought to be consistent with a blow from being hit by a pale during the alleged collapse of the bridge. No clear indication of interpersonal violence was found in these individuals.

In one skull from La Sarraz-Le Mormont, the mandible was missing but the cervical vertebrae were found in correct anatomical position. Cutting marks indicated that the mandible was removed before the skull was deposited in a pit (Dietrich et al. 2007). One possible explanation is that this was a battle trophy. Historical texts as well as archeological findings suggest that the heads of fallen enemies were cut off and kept by the Gaulois. Trophies are said to have been attached to the horses’ necks, nailed to houses, or embalmed and kept in cases (Dietrich et al. 2007).

### Isotopic data on diet and mobility

The last decade has seen a sharp increase of isotopic reconstructions of diet and mobility in Swiss archeological contexts dating to the Iron Age. Published data are available mainly for the Southern, Central, and Northern regions. Overall, isotopic data point to these populations having a mixed diet including both plants and animal (especially terrestrial) proteins and to the exploitation of both C_3_ and (to a lesser extent) C_4_ plants (Bucher et al. 2019; Knipper et al. 2014; Moghaddam et al. 2016; Moghaddam et al. 2018). Regional differences separate the Alpine regions from the Swiss Plateau, with the population inhabiting the latter featuring a diet with a higher contribution of C_4_ plants (likely millet) and animal proteins (Moghaddam et al. 2018).

Sex-related dietary differences have been detected at Münsingen-Rain, where males consumed more meat and/or dairy products than females, especially those individuals who had been buried with weapons (Moghaddam et al. 2016). Conversely, at Basel-Gasfabrik, the diets of males and females did not differ significantly (Knipper et al. 2017), and no straightforward relationships was found between isotopic and mortuary data.

Currently, regional mobility has been investigated only at two Iron Age sites: Münsingen-Rain (Hauschild et al. 2013; Moghaddam et al. 2016) and Basel-Gasfabrik (Knipper et al. 2018). Results suggest a frequency of nonlocals between 10 and 14.7% at Münsingen with no specific sex distribution. A different situation is represented by Basel-Gasfabrik, where the nonlocal individuals reach a frequency of 37% and include especially females.

## Discussion

### Representation

Many Late Iron Age sites were discovered and excavated in the nineteenth or early twentieth century. At this time, human remains were not a research and collection priority. Therefore, large numbers of skeletons were neither documented nor kept, while others were too poorly preserved for any statements to be made (Kaenel 1999). Even when human remains were collected during early excavations, generally only selected skeletal elements were kept. When the number of preserved skeletons is compared to the number of burials that were originally present and are known for some sites, it becomes evident that thousands of skeletons must have been destroyed or were not collected during early excavations.

As mentioned above, most Late Iron Age sites in Switzerland, and thus also the human remains, are concentrated around a few centers. It can be speculated to which degree this illustrates the actual situation in the Late Iron Age. At least partly, these concentrations may reflect areas of more intense archeological research both in the past and present.

### Funerary rites

In sum, while individual burials in cemeteries are generally considered the “normal” and by far most common case, this idea might have to be challenged for the Late Iron Age. Indeed, the variegated handling of the dead is a striking characteristic of the La Tène period, in the Swiss territory, and elsewhere in Europe. Like for the Swiss findings, several different interpretations for such “irregular” contexts have been discussed for sites in Austria, France, and Germany depending on their characteristics (Brunaux and Malagoli 2003; Hahn 1999; Lange 1995; Pichler et al. 2013; Teschler-Nicola 2017). They include multi-level funerary rites that may have included de-fleshing or exposure of corpses to birds and other animals, sacrifices and other ritual practices, and trophy hunting.

The interpretation of the human remains from the “irregular” contexts presented in this work is challenging in some cases because of the early and undocumented excavations. The completion of the ongoing work on skeletons and isolated bones from La Sarraz-Le Mormont and Basel-Gasfabrik holds potential for advancing the understanding of the funerary rites in contexts other than cemeteries.

### Demographic patterns

The high proportions of nonadults in Neolithic, Bronze Age, and High/Late Medieval contexts fit expectations for pre-modern attritional demographic profiles (Chamberlain 2006).

The La Tène, Roman, and Early medieval samples deviate from this trend and show a clear underrepresentation of nonadults. Excluding a low infant mortality for these periods (an explanation not supported by available archeological and historical data), we can try to isolate other factors responsible for this apparent demographic anomaly.

In Switzerland, only few neonate and infant skeletons were found in Roman necropolises, but hundreds have been excavated in settlement contexts (Grezet 2020; Kramis and Trancik 2014). This would correct the biased demographic profile from the necropolises used in this study and is consistent with what is known from classic sources (e.g., Cicero—De Legibus 2, 58; Juvenal—Saturae 15, 139; Fulgentius—Sermones Antiqui 7; Plinius—Naturalis Historia 7, 72), according to which children up to ca. 40 days old—or before they teethed—were not cremated but buried *intra muros*.

The demographic profile of the Roman period closely resembles that of early medieval samples. The underrepresentation of small children, especially neonates and small infants, in early medieval cemeteries (“Kleinkinderdefizit”) is a well-known phenomenon and has been discussed by many authors (Lohrke 2002). It is usually explained as the result of selective funerary practices (in particular, separate burial places for unbaptized children), and/or the effect of taphonomic and excavation-related damage to the fragile skeletal remains of the younger subadults. Early medieval settlements have hardly been excavated in Switzerland. This opens the intriguing possibility that during this period young subadults may also have been buried in domestic contexts. It has been hypothesized that the Roman custom of burying neonates in settlements may have its roots in the Iron Age (Langenegger 1996). Indeed, neonates have been found in Early Iron Age settlements in Brig-Glis/Waldmatte (Curdy et al. 1993) and in Lausanne (Langenegger 1996). Evidence exists from the Late Iron Age as well. The recent excavations at Basel-Gasfabrik have brought to light large numbers of neonate and infant skeletons in both the cemeteries and the settlement area (Pichler et al. 2015; Pichler et al. 2013; Rissanen et al. 2013). Altogether, the deficit of small children in the La Tène sample is reflected in the Roman and early medieval samples. Burials of small children in the settlement area at Basel-Gasfabrik give further weight to the theory of a special funerary treatment in the La Tène period, and the completion of the work on this site will possibly shed more light on this. Altogether, the findings suggest a possible continuity over centuries in the special funerary treatment of neonates and small infants, even though the beliefs behind the custom changed over time.

When attempting to interpret the results, several factors must however be kept in mind. Firstly, we considered only inhumations in this study, but in some periods, cremation was predominant (Late Bronze Age, Early Iron Age, and Antiquity). This leads to small sample sizes as well as a possible bias if selection mechanisms were applied with regard to the individuals who were either cremated or inhumated. Especially the Late Bronze and Early Iron Age are practically not represented in our sample.

Excavation-related factors could also be partly responsible for the deficit of the youngest age group. It is noteworthy that the neonate skeletons in the La Tène sample were found at sites that were excavated after the second half of the twentieth century. Since the human remains were generally not paid much attention to during early excavations, it is possible that they were overlooked, especially if no grave goods were associated with them. The numerous neonate skeletons in settlements from the Antiquity were all discovered in more recent excavations from the 1980s onward as well, owing to more refined excavation methods (Kramis and Trancik 2014). Large numbers of fetal, neonate, and infant bones at Basel-Gasfabrik were discovered only during the archeozoological analyses and had apparently not been recognized during the excavation (Pichler et al. 2013; Rissanen et al. 2013). Altogether, these findings suggest that the underrepresentation of children in the La Tène sample is strongly influenced by excavation-related factors besides possible selective funerary customs.

Regarding the possible relationship between demography and type of deposition, it is interesting to note that the sex ratio in irregular burials strongly deviates from that of “formal” funerary areas. Specifically, whereas the latter include an equal proportion of males and females (both 50% of sexed individuals), the proportion of the two sexes in irregular contexts is quite unbalanced (females 4.9%, males 39.3%). Even considering that a large number of individuals could not be assigned to a sex (39.3%), it is unlikely that this difference is purely due to chance. Age-wise, subadults are more frequent in regular (25.2%) than in irregular contexts (16.4%).

The findings in Switzerland suggest a selection mechanism (or several) that favored adult males for burial in irregular contexts. Similar observations have been reported from e.g. Austria (Teschler-Nicola 2017). Although the meaning of these contexts is not clarified, the high proportion of adult males may suggest a link to a high status of the deceased. Special offerings have often been found to be associated with such burials, e.g., in Basel-Gasfabrik and La Sarraz-Le Mormont (Dietrich et al. 2007; Hüglin and Spichtig 2010; Schaer and Stopp 2005). There are also clues from isotopic studies that suggested a possible higher social status of men (Moghaddam et al. 2016). Warfare events and associated trophy hunting may be another background of this observation (Hofeneder 2005; Müller 2009).

### Paleopathology

The observed adult prevalence of Cribra orbitalia is consistent with findings in later periods. A higher prevalence in children than in adults is also characteristic. The latter is due to Cribra orbitalia primarily reflecting stress phases in childhood and due to the fact that the lesions can remodel during adulthood. The conditions leading to these lesions have a negative impact on survivorship (Lewis 2007; Mittler and Van Gerven 1994; Papathanasiou et al. 2018). However, far-reaching conclusions about the living conditions can hardly be drawn based on such a small sample.

Our data suggest that highly cariogenic foods were not a major part of the diet during the La Tène period. While analyses of stable isotope ratios showed that the C_3_ plant-based diet might have been high in carbohydrates, it appears that it was not highly cariogenic, possibly because of low sugar content and infrequent meals. Moreover, the pronounced occlusal attrition observable for this period suggests abrasive foods, which could have had a natural cleaning effect and removed fissure caries. Groups with high attrition rates generally have low caries rates, even though the relationship between the two is not entirely clear (Hillson 1996).

The examination of traumata reveals clear differences between “regular” and “irregular” burials despite the difficulties arising from commingled remains and a lack of detail given in the original reports. Postcranial fractures that were most likely the result of falls and other accidents were found in both contexts. They affected males and females in similar proportions and were only found in adults. In cemeteries, cranial trauma was found in a small percentage of individuals. The affected individuals were all adult and in all but one case male. The proportion of skulls with traumatic lesions, which were without exception peri-mortem or post-mortem, was much higher in irregular burial sites, and both sexes in adults as well as children were affected. While the cranial traumata in cemeteries were most likely largely due to interpersonal violence, the picture in the sites with irregular burials was more ambiguous because many of the lesions were thought to represent post-mortem manipulations.

### Diet and mobility

Published isotopic studies for the Swiss Iron Age are still spatially biased (Knipper et al. 2018; Knipper et al. 2017; Moghaddam et al. 2016; Moghaddam et al. 2018), with the midlands being rather overrepresented. The picture emerging is however rather intriguing and raises several questions for future studies. Three aspects seem to deserve special attention: (a) the presence of regional differences in diet composition, (b) the possible association between social differentiation and diet, and (c) the presence of residential rules shaping mobility patterns.

The available data point to a marked variability across contexts in the relative exploitation of C_3_ vs. C_4_ plants, terrestrial vs. freshwater animal food sources, and dietary contribution of animal vs. plant proteins (Knipper et al. 2017; Moghaddam et al. 2016; Moghaddam et al. 2018). These results suggest important cultural, economic, and ecological differences across these “Celtic” populations and, potentially, their variable exposure to the Mediterranean economic sphere (Moghaddam et al. 2018). Strictly related is the type of relationship linking social differentiation and differential access to food resources. A simplistic model would expect high-quality diets for individuals enjoying a privileged social status, based on either status, sex, or a combination of these. We now know that things are actually more complex. This is clearly demonstrated by the diverging results obtained by published comparisons of paleodietary and funerary data (Laffranchi et al. 2019; Le Huray and Schutkowski 2005; Milella et al. 2019). Estimates of social differences based on funerary patterns are notoriously challenging (Parker Pearson 1982, 1993). Isotopic data, moreover, can mask subtle dietary differences of potential social relevance (e.g., the consumption of various cuts of meat and diverse types of animal products). As already mentioned, for the region and time under study, a dietary variability potentially linked to social differences has been highlighted only at Münsingen-Rain (Moghaddam et al. 2016). The fact that the same pattern was not observed in other contexts raises the possibility that on the Swiss territory and during the Late Iron Age, social differences (both vertical and horizontal) were multifaceted, and manifested variably.

A similar variability may also characterize the type of social factors influencing human mobility. The larger frequency of females among nonlocals at Basel-Gasfabrik has been interpreted by Knipper et al. (2018) as the evidence of a patrilocal residential system. Conversely, no such sex bias has been observed at Münsingen-Rain (Moghaddam et al. 2016), where nonlocals are, moreover, less numerous. The actual meanings of these differences are difficult to test, especially given the paucity of contexts whose isotopic data are directly comparable. The social, economic, and political factors behind mobility were likely numerous and intermingled with individual choices, in the past as nowadays (Anthony 1990; Burmeister 2000; De Ligt and Tacoma 2016; Eckardt 2010; Kearney 1986; Lee 1966; Massey et al. 1993).

## Conclusion

A review of anthropological, funerary, and biogeochemical data from the Late Iron Age in Switzerland is a challenging undertaking. In addition to the difficulties associated with hardly documented early excavations, incompletely collected skeletons, and poor skeletal preservation, further challenges arise from the quality and quantity of available anthropological data and its manner of publication. Findings are often published in different local languages, in a rather fragmented manner, or in inaccessible journals, and are often limited to age and sex determination. This has begun to change in recent years. Some human remains have been subject to intense anthropological research that has shed light on aspects such as diet and mobility or on selected burials. However, systematic osteological or paleopathological studies are still almost non-existent, and the interpretation of the available results is often hampered by purely descriptive approaches, methodological inconsistencies, and lack of detail in original reports.

Nevertheless, we tried to draw a general picture of the anthropological findings in skeletons from the Late Iron Age and identified aspects worthy of further research. The demographic findings revealed that children are strongly underrepresented in the skeletal record of this period, and the comparison with samples from other periods indicated that this might be the result of different funerary rites (particularly burial in separate locations) for the youngest age groups. Furthermore, differences were found between regular and irregular burial sites, with the latter exhibiting a predominance of adult males but fewer adult females and children.

Regular and irregular burial sites also differed with regard to the prevalence of cranial trauma. In the irregular burial sites, a large proportion of individuals exhibited peri-mortem or post-mortem traumata that were not identified in regular cemeteries.

We identified research potential especially for paleomobility, for which only two sites have been published to date, and for systematic paleopathological studies. The prevalence and patterning of trauma, markers of unspecific stress, and dental pathologies are only some aspects that merit further consideration. Pathological alterations indicative of infectious disease have not been reported for any of the individuals in the sample. This raises the question whether markers of infectious disease were not present or if they were not recorded or were not recognizable due to poor preservation or other factors.

The recent excavations at La Sarraz-Le Mormont and Basel-Gasfabrik, among others, offer the opportunity to study well-documented assemblages, which may greatly enhance the knowledge of the funerary customs in a sanctuary as well as in a settlement and its necropolises.

## Data Availability

All data collected are published within this article or in the literature cited.
